# Efficacy of the Combination of λ-Cyhalothrin and Chlorantraniliprole Against Four Key Storage Pests

**DOI:** 10.3390/insects16040387

**Published:** 2025-04-04

**Authors:** Waqas Wakil, Nickolas G. Kavallieratos, Aqsa Naeem, Hamza Jamil, Demeter Lorentha S. Gidari, Maria C. Boukouvala

**Affiliations:** 1 Department of Entomology, University of Agriculture, Faisalabad 38040, Pakistan; aqsanaeem231@gmail.com (A.N.); hamzajamil4800@gmail.com (H.J.); 2 Senckenberg German Entomological Institute, D-15374 Müncheberg, Germany; 3 Laboratory of Agricultural Zoology and Entomology, Department of Crop Science, Agricultural University of Athens, 75 Iera Odos Str., 11855 Athens, Greece; dlgidari@aua.gr (D.L.S.G.); mbouk@aua.gr (M.C.B.)

**Keywords:** λ-cyhalothrin, chlorantraniliprole, stored-product pests, persistence, progeny, commodities, mortality

## Abstract

With over 1000 species of pests causing losses in both the quantity and quality of stored food, insect contamination poses significant challenges. The present study assesses the efficacy of the combination of λ-cyhalothrin and chlorantraniliprole against four key storage pests—*Trogoderma granarium*, *Sitophilus oryzae*, *Rhyzopertha dominica*, and *Tribolium castaneum*. Laboratory bioassays demonstrated species-dependent mortality, with *S. oryzae* and *R. dominica* suffering 100% mortality in several tested scenarios. A 90-day persistence trial revealed decreased efficacy over time, especially for *T. granarium* (32.0–71.4% at 0 days and 0.0–7.5% at 90 days) and *T. castaneum* (38.8–82.7% at 0 days and 0.0–12.7% at 90 days) vs. *S. oryzae* and *R. dominica*. Progeny production of *S. oryzae* and *R. dominica* was almost suppressed in persistence trials (0.4 individuals per vial and 1 individual per vial, respectively) after 30 days of storage at the dose of 5 mg/kg wheat. The results highlight the variability in insecticidal performance based on species, dose, exposure, and commodity type, emphasizing the need for tailored pest management strategies in the storage environment.

## Simple Summary

The present study evaluated the effectiveness of the combination of λ-cyhalothrin and chlorantraniliprole against *Trogoderma granarium*, *Tribolium castaneum*, *Rhyzopertha dominica*, and *Sitophilus oryzae*, on treated wheat, rice, and maize. *Sitophilus oryzae* and *R. dominica* were the most susceptible species in both laboratory and persistence trials. The progeny production of *S. oryzae* and *R. dominica* was almost suppressed in laboratory trials on 5 ppm-treated wheat and persistence bioassays, 30 days post-storage. *Trogoderma granarium* and *T. castaneum* were less affected by the tested insecticidal combination, suffering lower mortality rates and producing higher progeny, compared to *S. oryzae* and *R. dominica*. Concerning the different commodities, treated wheat led to higher mortalities and lower progeny vs. rice and maize. These findings demonstrate that the combination of λ-cyhalothrin and chlorantraniliprole is effective under testing of different parameters, such as the type of the grain commodity or insect species.

## 1. Introduction

Insect infestation in food industries is a serious issue of concern. Over a thousand species of pests attack stored products, causing both qualitative and quantitative losses (9% in developed countries while >20% in developing countries) in storage facilities [1,2]. Among them, coleopterans such as the dermestid *Trogoderma granarium* (Everts), the bostrychid *Rhyzopertha dominica* (F.), the tenebrionid *Tribolium castaneum* (Herbst), and the curculionid *Sitophilus oryzae* (L.) cause several types of damages in wheat, sorghum, maize, barley, rice and other stored grains [3,4,5]. All 4 species belong to the 7 most destructive species out of the 18 listed stored grain insect pests in Punjab, Pakistan [6].

*Trogoderma granarium*, one of the most damaging pests of stored grains, degrades grains and other commodities, rendering the infested products unfit for human consumption. It can be easily spread through the movement of products around the world, lying undetected as adults, eggs, and larvae (diapausing or not). To prevent its importation, several countries have strong quarantine laws in place, and exports from countries with established populations are subjected to limitations and controls [7]. *Rhyzopertha dominica* is a primary pest of cereal commodities in many regions of the world, including tropical and subtropical regions [8,9]. The direct feeding activity of the voracious larvae and adults on the germ and endosperm of the cereal leads to elevated grain losses [10]. The cosmopolitan stored-product pest *T. castaneum* has the capacity to infest 246 grain commodities [2,11], causing severe economic losses. For instance, it causes up to 50% of the economic losses of cocoa beans worldwide [12]. *Sitophilus oryzae* is one of the most important species of stored products [2,13] and is difficult to control. This is because the immature stages develop inside grain kernels, which hinders the accurate detection of infestations and the effectiveness of control measures [3].

The halogenated type II pyrethroid λ-cyhalothrin [14] is recognized as an effective insecticide against numerous insect species, including stored-product pests [15,16,17,18,19]. It acts through the neurotoxic action on the sensory nerves, altering the passage characteristics of voltage-sensitive sodium channels [20]. It also interferes in calcium and chloride channels, disrupting further nerve activity. It quickly impairs muscular control, prevents food consumption, and leads to death, since it can easily be absorbed by biological tissues and penetrates the cuticle of the insect [21,22,23].

Anthranilic diamide insecticides are novel systemic insecticides. They act by enhancing the uncontrollably released calcium in insects through the activation of their ryanodine receptor, leading to rapid muscle disfunction, paralysis, and death [24,25]. Among them, chlorantraniliprole exhibits a low toxicity to mammals and is effective in controlling numerous insect pests without endangering beneficial species [24,26,27,28]. Furthermore, chlorantraniliprole has low intrinsic toxicity towards beneficial arthropods and non-target species, like earthworms, honeybees, and other pollinators [29,30,31,32,33]. Concerning λ-cyhalothrin, it is toxic to honeybees, demonstrating side effects even in sublethal concentrations [34,35]. Nevertheless, λ-cyhalothrin exhibited a low suppression rate on soil microbial respiration, which fully recovered in less than one month [36].

Pyrethroids, carbamates, and organophosphates are among the various insecticide types that have been widely used in the protection of stored products [37]. Previous investigations have demonstrated their long-term residual activity against a broad range of stored-product species [38,39,40,41,42]. However, because of the prolonged and continuous use of numerous insecticidal active ingredients, many species have developed resistance, leading to inadequate utilization [43,44,45]. Alternative management strategies have been used to deal with this developed resistance, such as combining insecticides with various modes of action. For instance, Nayak and Daglish [46] revealed that the spinosad + chlorpyrifos-methyl combination provided complete control of four storage psocid pests belonging to genus *Liposcelis*. The residual efficacy of aerosols containing 1% pyrethrin + methoprene and 3% pyrethrin + methoprene was tested against *T. castaneum* and *Tribolium confusum* Jacquelin du Val (Coleoptera: Tenebrionidae). The results show that both combinations achieved the residual control of both species, with *T. castaneum* being the most susceptible [47]. Moreover, the combination of chlorpyrifos-methyl (3 ppm) and deltamethrin (0.5 ppm) achieved control on *R. dominica* and *T. castaneum* adults both resistant and susceptible to phosphine [48]. Additionally, Wakil et al. [49] examined the application of the corresponding combined pairs of *Metarhizium robertsii* J.F. Bisch., Rehner & Humber (Clavicipitaceae), an entomopathogenic fungus (EPF), the Protect-It diatomaceous earth (DE), and λ-cyhalothrin against *R. dominica*, *T. castaneum*, and *T. granarium*. For all examined insect species, the combination of the DE + λ-cyhalothrin was the most effective treatment of mortality in laboratory trials on persistence and progeny production, followed by the combinations of the entomopathogenic fungi + λ-cyhalothrin and entomopathogenic fungi + DE.

Both λ-cyhalothrin and chlorantraniliprole have been evaluated against various stored product species belonging to different orders, such as Coleoptera, Lepidoptera, and Psocoptera [19,50,51,52,53]. However, there are no data concerning their combined use against stored-product pests. The current study evaluates the efficacy of a λ-cyhalothrin + chlorantraniliprole combination against four serious insect pests in storage facilities under various parameters, i.e., commodities, doses, and exposure intervals.

## 2. Materials and Methods

### 2.1. Tested Commodities

All commodities used for the trials were free of infestation and insecticides. *Oryza sativa* L. (var. Kainat Basmati) rice, *Zea mays* L. (var. DK-6525) maize, and *Triticum aestivum* L. (var. Noor 2013) wheat were the commodities being tested. Rice (11.2%), maize (12.5%), and wheat (11.5%) were evaluated for their moisture content using the GAC 2700-AGRI (Dickey-John Co., Auburn, IL, USA) moisture meter. The commodities under trial were sieved to eliminate contaminants and dockage before any treatment.

### 2.2. Tested Insects

The Microbial Control Laboratory at the University of Agriculture, Faisalabad, provided the insect populations used in this study. For more than a decade, these insect populations had been reared without any contact with insecticides, at 30 °C, in 65% relative humidity (RH) and total darkness. Apart from *T. castaneum*, which was reared on wheat flour with 5% brewer’s yeast, the other three insect species were reared on whole wheat. For *T. granarium*, adults under 24 h old and of mixed sex were used. Adults of *T. castaneum*, *S. oryzae*, and *R. dominica* (of mixed sex) younger than two weeks were also used [54].

### 2.3. Insecticide

Laboratory and persistence treatment bioassays were conducted using Ampligo 150 ZC (Syngenta, Karachi, Punjab, Pakistan), a suspension concentrate (SC) that contained 50 g/L λ-cyhalothrin and 100 g/L chlorantraniliprole.

### 2.4. Laboratory Bioassays

The combination of λ-cyhalothrin + chlorantraniliprole was used in solution form at a dose rate of 0.01 mg/kg grain (containing 0.0005 mg λ-cyhalothrin and 0.001 mg chlorantraniliprole), 0.1 mg/kg grain (containing 0.005 mg λ-cyhalothrin and 0.01 mg chlorantraniliprole), 1 mg/kg grain (containing 0.05 mg λ-cyhalothrin and 0.1 mg chlorantraniliprole), and 5 mg/kg grain (containing 0.25 mg λ-cyhalothrin and 0.5 mg chlorantraniliprole), based on doses previously used [49,50]. Using trays, 1000 g of each grain, spread out on a tray, was sprayed with a 1 mL aqueous solution that contained the appropriate quantity of the insecticide for each dose. The spraying process was carried out with the help of a G233 airbrush (Master Airbrush, North Las Vegas, NV, USA). Additionally, a control group for each tested commodity was submitted to 1 mL distilled water treatment by using another airbrush reserved for the control groups. For each process of spraying, different trays were cast off. Each treated grain stack was kept separate in glass jars of 5 L and shaken (by hand) for at least 10 min to attain a uniform distribution of tested insecticide on all grain mass [55]. From each untreated or treated lot, a triad of samples weighing 20 g was placed in glass vials that were 7.5 cm in diameter and 12.5 cm in height. Each glass jar included a separate scoop that was used to collect samples. An ELB 300 e-balance (Shimadzu, Kyoto, Japan) was used to weigh the samples on a fine paper layer. Every weighing session used a new paper layer. At the closure of each vial, there was a gauze-covered hole of 1.5 cm in diameter to provide adequate aeration. Every single vial contained 50 adults of each species. To prevent the individuals from absconding, a layer of polytetrafluoroethylene (Sigma-Aldrich Chemie GmbH, Taufkirchen, Germany) entirely covered the inside neck of the glass vials. The vials were maintained in incubators with 65% RH and 30 °C throughout the entire experiment. The adult mortality of each insect species was determined by prodding each insect gently with a different brush, per dose/formulation and control, under a Leica stereomicroscope (Wild M3B, Heerbrugg, Switzerland) after 7 and 14 days of exposure. For every exposure interval, different individuals, commodities, and vials were used. The progeny production of all tested insects was also assessed. After the 14-day mortality count, all individuals contained in vials were discarded, and all vials were reinserted into the incubators. The offspring production was assessed after 62 days for *R*. *dominica* and *T. castaneum*, 46 days for *T. granarium*, and 60 days for *S. oryzae* [56,57]. For *T. granarium* and *T. castaneum*, immature stages and adults were recorded for the evaluation of progeny, while for *R. dominica* and *S. oryzae*, only adults were taken under observation, since their immature stages grow inside kernels [2]. Three iterations of the trials were conducted by using new individuals, commodities, and vials.

### 2.5. Persistance Bioassays

Throughout a period of three months, four laboratory trials (0, 30, 60, and 90 days) were conducted to assess the effects of four dose treatments in three commodities as well as the control groups, at 30 °C and 65% RH, as mentioned above. The glass jars under trial were kept in the incubator at the same temperature and RH for the entire observational period [54,57]. After seven days of exposure to the insecticide, the mortality rates of every insect were assessed, and the production of offspring was evaluated as previously mentioned.

### 2.6. Statistical Analysis

The control mortality was low (<5%) for all tested species. For laboratory mortalities, an Abbott’s correcting mortality formula was used [58]. Before analysis, all data were converted to log (x + 1) [59] for normal variance. All data were subjected to 3-way analysis of variance (ANOVA), with exposure interval, dose rate and commodities as main effects, and mortality as the response variable. Interactions among all main effects were also considered in the analysis. Similarly, all data of progeny production for laboratory trials were subjected to 3-way ANOVA. For progeny production, the main effects were dose rates, commodities and insect species, while progeny number was the response variable. The interactions of all main effects in progeny were also included during analysis. To make a comparison among means in progeny and mortality, a Tukey (HSD) test at *p* = 0.05 was performed [60]. In the case of persistence bioassays, the Abbott’s formula was used for the correction of mortality [58]. The data were converted to log (x + 1) to regulate the variance prior to analysis [59]. Data were subjected to 2-way ANOVA for each tested insect species. For analysis, all main effects (period of storage and dose rate) and response variables (mortality) along with their associated interactions were considered. For the persistence of progeny production, all main effects (period of storage and dose rate) and the response variable (progeny number) along with their associated interactions were subjected to a 2-way ANOVA. Tukey HSD test set at *p* = 0.05 was used to compare the means in mortality and progeny production [60].

## 3. Results

### 3.1. Mortality in Laboratory Bioassays

For all tested species, all main effects were significant (Table 1). The commodity × exposure interval interaction was not significant for *R. dominica*, while commodity × dose rate and exposure interval × dose rate interactions were significant for *R. dominica* and *T. granarium*, respectively. Commodity × exposure interval × dose rate interaction was significant for *S. oryzae* and *R. dominica* (Table 1). Among all commodities, the highest mortality rates were obtained on treated wheat for all tested species, regardless of time interval and dose rate (Table 2). The dose of 5 mg/kg grain of the formulation achieved complete mortality in *S. oryzae* and *R. dominica* adults on treated wheat and rice, 14 days post-exposure, while maize led to 94.8 and 91.1% mortality, respectively. The second highest mortality rate (95.9%) has been noted for *T. castaneum* at 5 mg/kg grain-treated wheat after 14 days of exposure. However, rice and maize scored mortality rates <85%. Regarding *T. granarium*, the highest mortality rate observed on 5 mg/kg grain-treated wheat did not exceed 88% after 14 days of exposure (Table 2).

### 3.2. Progeny in Laboratory Bioassays

For the progeny production, all main effects along with their associated interactions were significant (Table 3). The progeny production of *S. oryzae* was <1 individual/vial on 1 mg/kg grain-treated wheat and 5 mg/kg grain-treated wheat and rice. Regardless of the dose rate, treated maize led to higher progeny production compared to the other commodities. A remarkable reduction in progeny production was noted for *R. dominica* on treated wheat and rice compared to the respective control groups. The progeny production number was less than 1 individual/vial for *R. dominica* on 5 mg/kg grain-treated wheat. The progeny production of *T. castaneum* was significantly reduced, even at 0.01 mg/kg grain of the formulation on all treated commodities, compared to the control groups (28.0 vs. 88.6 individuals/vial on wheat, 33.7 vs. 64.1 individuals/vial on maize, and 20.1 vs. 39.4 individuals/vial on rice). Concerning *T. granarium* and regardless of the dose rate or the treated grain, progeny production was noted to be the least affected among all tested species (Table 4).

### 3.3. Mortality in Persistence Bioassays

In laboratory persistence trials and seven days post-exposure, all main effects along with their associated interactions were significant for all tested species (Table 5). The highest mortality rates were noted for *S. oryzae* and *R. dominica* at a 5 mg/kg grain dose at 0 days of storage, achieving 94.2 and 87.4%, respectively. After 30 days of storage at 5 mg/kg grain dose, mortality rates were significantly reduced but remained higher than 60% (68.7 and 61.3%, respectively). Lower mortality rates were noted for *T. castaneum* and *T. granarium* during the whole period of storage, with values of 53.6 and 46.8%, respectively, at a 5 mg/kg grain dose after 30 days of storage (Table 6).

### 3.4. Progeny in Persistence Bioassays

All main effects along with their interactions significantly impacted progeny production in persistence bioassays (Table 7). The dose of 5 mg/kg grain almost suppressed progeny production of all four tested species at 0 days (0.4–0.9 individuals per vial), and for *S. oryzae* or *R. dominica* at 30 days (0.4 and 1.0 individuals per vial, respectively). Also, for *S. oryzae*, the dose of 1 mg/kg grain-treated wheat at 0 and 30 days of storage almost suppressed progeny production. Regarding *T. granarium*, at 5 mg/kg grain dose rate, the progeny mean number reached 9.7 individuals per vial at 30 days of storage but progressively increased to 23.9 individuals per vial at 90 days. The progeny production of *T. castaneum* ranged from 3.1 to 17.7 (5 mg/kg grain) (Table 8).

## 4. Discussion

The current study offers various biotic and abiotic parameter-dependent results. λ-Cyhalothrin + chlorantraniliprole demonstrated high efficacy in controlling *S. oryzae* and *R. dominica*. *Tribolium castaneum* and *T. granarium* showed lower susceptibility, especially on maize and rice compared to wheat. Progeny production was significantly reduced for *S. oryzae* and *R. dominica* on all grains tested at higher doses (1 and 5 mg/kg grain) compared to the control groups. Regarding the persistence trials, mortality rates declined over time for all species, being more vigorous for *T. castaneum* and *T. granarium*. The suppression of progeny production for *S. oryzae* and *R. dominica* remained at 5 mg/kg grain after 30 days, although progeny increased at longer storage periods.

By examining λ-cyhalothrin at 1.25 mg a.i./kg wheat, Wakil et al. [49] reported 49.1, 41.7, and 57.2% mortalities for *T. castaneum*, *T. granarium*, and *R. dominica*, respectively, after 14 days of exposure. In contrast, the present study achieved 95.9, 87.3, and 100% mortalities, respectively, with just 0.25 mg a.i./kg in a combined insecticidal treatment. Similarly, for the progeny production, Wakil et al. [49] recorded 49.6, 72.5, and 56.3 individuals/vial, which values are notably higher compared to the current study (7.7, 13.5, and 0.7 individuals/vial). Chlorantraniliprole at 1 mg a.i./kg (WG and SC formulations) caused 63.3 and 36.7% mortality in *R. dominica*, respectively, and 71.1 and 70.1% in *S. oryzae*, respectively, after 14 days of exposure [50]. In contrast, using half that dose, the tested combination of the present study achieved 100% mortality of both species. Regarding progeny production, Kavallieratos et al. [50] recorded no fewer than eight and one individuals/vial for *R. dominica* and *S. oryzae*, respectively, whereas in this study, the progeny productions of both species were almost suppressed (less than one individual/vial). Based on those previous studies, it becomes evident that both λ-cyhalothrin and chlorantraniliprole are effective against various stored-product pests at specific dose rates. Interestingly, the current study highlighted that the tested combination is effective at remarkably lower concentrations, suggesting also that their combination exhibits additive effects against *S. oryzae* and *R. dominica*. In several cases, when pyrethroids are combined with organophosphate insecticides, they lead to additive or synergistic effects [61,62,63,64,65]. In the present case, the exact mode of action of the combination of the pyrethroid λ-cyhalothrin and the anthranilic diamide chlorantraniliprole has not been investigated. Generally, when one insecticide in the mixture obstructs the metabolic detoxification of the other insecticide, the toxicity of the latter is increased, explaining the additive effects of the insecticidal combinations [65,66].

The control of *S. oryzae* and *R. dominica* is often challenging as most of their developmental stages grow inside the kernels [67]. Females of *R. dominica* deposit eggs on the surface of the grain [8], and the first-instar larva either penetrates the outer surface directly or enters through a crack or opening consuming the kernel. Females of *S. oryzae* oviposit inside the kernel, where all the developmental stages grow [67]. A very compelling result of the current study concerns the complete mortality of *S. oryzae* and *R. dominica* at 5 mg formulation/kg wheat or rice 14 days post-exposure, along with their almost suppressed progeny production, as well as their susceptibility in persistence trials. Concerning *T. castaneum* and *T. granarium*, even though their mortality rates did not exceed 55%, the progeny production remained at <10 individuals/vial. The high mortalities of *S. oryzae* and *R. dominica* are linked with the suppression of offspring production vs. the lower mortalities of *T. castaneum* and *T. granarium*, and higher progeny. Probably, the tested insecticidal combination prohibited female individuals of *S. oryzae* and *R. dominica* from oviposition before their death through the neurotoxic activity of the pyrethroid and the paralytic action of the anthranilic diamide, leading to a reduction in offspring emergence. Whether the mechanisms of action of the tested insecticides operate independently or are combined when they are simultaneously present in the insects’ organism remains to be investigated. A previous study evaluating thiamethoxam and chlorantraniliprole combinations revealed that after 30 days of storage, *S. oryzae* progeny was suppressed on wheat, rice, and maize at various combinations of doses, while mortality remained below 85% [54]. The same study showed that 1 mg thiamethoxam/kg plus 0.5 mg chlorantraniliprole/kg and 5 mg thiamethoxam/kg plus 2.5 mg chlorantraniliprole/kg completely prevented the progeny emergence of *R. dominica*, though mortality did not exceed 74% after the same storage period. For *T. castaneum* and *T. granarium*, mortality remained below 64% at the higher doses, but progeny production was reduced to <5 individuals/vial, which is significantly lower compared to >105 in the control groups [54]. The results of Wakil et al. [54] and the current study prove that chlorantraniliprole, in combination with another type of insecticide, is efficient against specific stored-product species, such as *S. oryzae* and *R. dominica*. This study also points out that the combined use of chlorantraniliprole with λ-cyhalothrin was not equally effective against all tested species. This is because *T. castaneum* and *T. granarium* were tolerant to the combined treatments tested. Obviously, there are barriers that prevent an elevated activity of the examined combination against a broad spectrum of insects. It is well known that several CYP genes (cytochrome P450) in *T. castaneum* are rapidly upregulated under the pressure of insecticides. This issue is linked with the detoxification of insecticides offering *T. castaneum* tolerance to numerous compounds. For example, the genes CYP345A1 and CYP4G7 are significantly upregulated by pyrethroids, including λ-cyhalothrin (1.81- and 1.73-fold, respectively) [68]. To the best of our knowledge, there are no relevant data about upregulated genes in *T. castaneum* due to exposure to chlorantraniliprole. On the other hand, it is thought that combinations of insecticides could make a path to overcome the problem of resistance in stored-product insects [46,69]. However, this hypothesis has been only partially confirmed. For instance, Daglish [69] documented that a combination of the bacterial-based insecticide spinosad (1 mg/kg wheat) and the organophosphorus insecticide chlorpyrifos-methyl (10 mg/kg wheat) successfully managed insecticide-resistant strains of *T. castaneum*, *R. dominica*, *S. oryzae*, and *Cryptolestes ferrugineus* (Stephens) (Coleoptera: Laemophloeidae), but not *Oryzaephilus surinamensis* (L.) (Coleoptera: Silvanidae). Similarly, another combination of the same insecticides at 0.5 and 2.5 mg a.i./kg wheat, respectively, did not control *Liposcelis entomophila* (Enderlein) (Psocoptera: Liposcelididae) vs. another three *Liposcelis* spp., all resistant to various contact insecticides [46]. Therefore, to reinforce the findings of this study, the combination of chlorantraniliprole or λ-cyhalothrin with other types of insecticides needs to be investigated against different stored-product insect species, including strains resistant to insecticides.

The type of the treated commodity played a decisive role in the mortality results of the current study. The higher mortality rates were noted on treated wheat, followed by treated rice and maize. Kavallieratos et al. [50] evaluated chlorantraniliprole-treated maize, artificially peeled rice, barley, whole rice, hard wheat, and oats at different doses against adults of *Liposcelis bostrychophila* Badonnel (Psocoptera: Liposcelididae), larvae of *Ephestia kuehniella* Zeller (Lepidoptera: Pyralidae), adults of *R. dominica*, adults and larvae of *T. confusum*, and adults of *S. oryzae*. The results show that the effectiveness of the insecticide varied depending on the commodity, with treated maize leading to the lowest mortality rates of all tested species. The commodity-dependent efficacy of an insecticide is evident in numerous studies [70,71,72,73,74], indicating that the chemical characteristics of each type of grain can influence their effectiveness, as the insecticides show variation in their degradation or dissipation on different types of grains [75]. For instance, Chintzoglou et al. [75] observed accelerated degradation and differences in the rate dissipation of spinosad on maize, compared to barley and wheat, issues that could be linked to the lower mortalities of *R. dominica* and *S. oryzae* observed on maize.

## 5. Conclusions

To conclude, the combination of λ-cyhalothrin and chlorantraniliprole, along with different parameters, such as the type of commodity, is effective against various pests of stored products, even in long-term control, at notably lower doses compared to those that have been used for single application. The combinations of insecticides belonging to different classes could be further evaluated, targeting a considerable reduction in their doses. Additionally, the investigation of different combinations of abiotic parameters, such as temperature or surface of application, could lead to optimal and targeted protection strategies in the storage environment.

## Figures and Tables

**Table 1:** ANOVA parameters of main effects and associated interactions for mortality levels of *S. oryzae*, *T. castaneum, T. granarium*, and *R. dominica* adults under laboratory trials (total df = 215 for all species).

Species		*S. oryzae*		*T. castaneum*		*T. granarium*		*R. dominica*	
Source	df	*F*	*p*	*F*	*p*	*F*	*p*	*F*	*p*
Commodity	2	156.94	<0.01	142.63	<0.01	152.93	<0.01	150.67	<0.01
Exposure interval	1	732.38	<0.01	562.90	<0.01	663.12	<0.01	833.34	<0.01
Dose rate	3	614.09	<0.01	522.29	<0.01	400.75	<0.01	727.93	<0.01
Commodity × exposure interval	2	3.21	0.04	5.44	<0.01	3.15	0.04	0.03	0.97
Commodity × dose rate	6	1.40	0.22	1.74	0.11	0.80	0.56	2.32	0.03
Exposure interval × dose rate	3	1.48	0.22	2.07	0.10	3.32	0.02	0.87	0.45
Commodity × exposure interval × dose rate	6	9.85	<0.01	0.17	0.98	0.70	0.64	3.67	<0.01

**Table 2:** Mean mortality (% ± SE) of *S. oryzae, T. castaneum, T. granarium*, and *R. dominica* adults exposed for 7 and 14 days on three grain commodities treated with four dose rates of the combination of λ-cyhalothrin and chlorantraniliprole in laboratory trials. For each species within each commodity, means followed by the same lowercase letter are not significantly different; df = 3, 35, Tukey–Kramer (HSD) test at *p* = 0.05. For each species within each dose rate, means followed by the same uppercase letter are not significantly different; df = 2, 26, Tukey (HSD) test at *p* = 0.05.

		**Exposure Interval (7 Days)**					
		**Dose Rate (ppm)**					
**Species**	**Commodity**	**0.01**	**0.1**	**1**	**5**	***F***	***p***
*S. oryzae*	Wheat	47.46 ± 0.85 Ad	64.75 ± 1.73 Ac	78.94 ± 1.13 Ab	92.84 ± 1.18 Aa	235.2	<0.01
	Maize	39.85 ± 1.30 Bc	51.53 ± 2.03 Bb	62.19 ± 1.39 Ca	67.34 ± 1.26 Ca	63.5	<0.01
	Rice	43.88 ± 1.76 ABd	56.11 ± 1.96 Bc	69.78 ± 2.06 Bb	83.53 ± 2.01 Ba	76.5	<0.01
	*F*	7.83	12.3	28.2	70.5		
	*p*	<0.01	<0.01	<0.01	<0.01		
*T. castaneum*	Wheat	36.02 ± 1.35 Ad	49.34 ± 1.17 Ac	65.34 ± 1.65 Ab	74.09 ± 1.95 Aa	117.6	<0.01
	Maize	25.27 ± 2.38 Bd	41.60 ± 1.61 Bc	50.51 ± 1.15 Bb	59.02 ± 2.03 Ba	60.5	<0.01
	Rice	31.26 ± 2.13 ABd	46.74 ± 1.10 ABc	57.74 ± 1.49 Cb	68.38 ± 1.64 Aa	94.1	<0.01
	*F*	7.25	8.99	26.2	16.3		
	*p*	<0.01	<0.01	<0.01	<0.01		
*T. granarium*	Wheat	32.82 ± 2.12 Ac	42.10 ± 1.71 Ab	56.76 ± 2.29 Aa	63.04 ± 1.70 Aa	48.3	<0.01
	Maize	21.49 ± 1.33 Bc	31.38 ± 1.59 Bb	42.65 ± 1.25 Ba	47.78 ± 1.52 Ba	67.2	<0.01
	Rice	27.86 ± 1.83 ABd	37.34 ± 1.80 ABc	48.91 ± 2.21 Bb	59.80 ± 1.88 Aa	51.0	<0.01
	*F*	10.0	9.89	12.8	22.1		
	*p*	<0.01	<0.01	<0.01	<0.01		
*R. dominica*	Wheat	41.46 ± 1.26 Ad	57.79 ± 1.92 Ac	68.69 ± 1.39 Ab	86.03 ± 1.93 Aa	127.4	<0.01
	Maize	33.21 ± 1.57 Bd	45.86 ± 1.93 Bc	54.78 ± 1.22 Bb	65.77 ± 1.22 Ca	82.5	<0.01
	Rice	35.38 ± 2.54 Bd	51.18 ± 1.16 Bc	62.87 ± 2.03 Ab	78.33 ± 1.59 Ba	141.9	<0.01
	*F*	10.2	12.1	19.3	40.2		
	*p*	<0.01	<0.01	<0.01	<0.01		
			**Dose Rate (ppm)**				
			**Exposure Interval (14 Days)**				
**Species**	**Commodity**	**0.01**	**0.1**	**1**	**5**	***F***	***p***
*S. oryzae*	Wheat	73.29 ± 1.47 Ad	81.15 ± 2.08 Ac	90.42 ± 1.70 Ab	100.00 ± 0.00 Aa	56.7	<0.01
	Maize	54.81 ± 1.84 Cd	65.51 ± 1.79 Cc	82.41 ± 1.17 Bb	94.81 ± 1.59 Ba	119.1	<0.01
	Rice	61.38 ± 1.77 Bd	73.45 ± 1.22 Bc	87.58 ± 1.59 ABb	100.00 ± 0.00 Aa	157.8	<0.01
	*F*	30.2	20.3	7.25	10.6		
	*p*	<0.01	<0.01	<0.01	<0.01		
*T. castaneum*	Wheat	57.71 ± 2.08 Ad	66.67 ± 2.18 Ac	82.79 ± 1.70 Ab	95.89 ± 1.17 Aa	85.9	<0.01
	Maize	41.78 ± 1.75 Bd	53.42 ± 1.35 Bc	65.76 ± 0.93 Bb	76.35 ± 1.60 Ca	107.5	<0.01
	Rice	46.67 ± 1.58 Bd	59.55 ± 1.33 Bc	71.63 ± 2.13 Bb	83.73 ± 1.96 Ba	79.8	<0.01
	*F*	20.2	15.7	27.0	37.2		
	*p*	<0.01	<0.01	<0.01	<0.01		
*T. granarium*	Wheat	51.54 ± 1.53 Ad	62.56 ± 1.06 Ac	73.17 ± 1.55 Ab	87.29 ± 1.21 Aa	126.9	<0.01
	Maize	37.65 ± 1.86 Bd	46.93 ± 1.95 Cc	55.11 ± 1.39 Cb	69.55 ± 1.27 Ca	67.4	<0.01
	Rice	43.49 ± 1.50 Bd	54.10 ± 1.34 Bc	64.76 ± 1.78 Bb	76.36 ± 1.55 Ba	82.5	<0.01
	*F*	18.0	27.2	32.5	43.6		
	*p*	<0.01	<0.01	<0.01	<0.01		
*R. dominica*	Wheat	63.71 ± 2.09 Ad	78.42 ± 1.58 Ac	86.96 ± 1.82 Ab	100.00 ± 0.00 Aa	90.5	<0.01
	Maize	48.12 ± 1.04 Bd	61.53 ± 1.70 Bc	73.19 ± 1.49 Bb	91.09 ± 1.33 Ba	166	<0.01
	Rice	52.59 ± 2.37 Bd	67.48 ± 2.00 Bc	81.32 ± 1.48 Ab	100.00 ± 0.00 Aa	138	<0.01
	*F*	17.4	23.4	18.5	44.2		
	*p*	<0.01	<0.01	<0.01	<0.01		

**Table 3:** ANOVA parameters of main effects and associated interactions for progeny production of *S. oryzae, T. castaneum, T. granarium*, and *R. dominica* individuals in laboratory trials (total df = 539 for all species).

Source	df	*F*	*p*
Species	3	437.27	<0.01
Commodity	2	119.89	<0.01
Dose rate	4	5606.17	<0.01
Species × commodity	6	229.11	<0.01
Species × dose rate	12	70.80	<0.01
Commodity × dose rate	8	87.69	<0.01
Species × commodity × dose rate	24	71.98	<0.01

**Table 4:** Mean live numbers (± SE) of *S. oryzae, T. castaneum, T. granarium*, and *R. dominica* individuals following the exposure of parents to three grain commodities, treated with four dose rates of the combination of λ-cyhalothrin and chlorantraniliprole and untreated control (0 ppm) in laboratory trials. For each species within each commodity, means followed by the same lowercase letter are not significantly different; df = 4, 44, Tukey (HSD) test at *p* = 0.05. For each species within each dose rate, means followed by the same uppercase letter are not significantly different; df = 2, 26, Tukey (HSD) test at *p* = 0.05.

		Dose Rate (ppm)						
Species	Commodity	0	0.01	0.1	1	5	*F*	*p*
*S. oryzae*	Wheat	69.48 ± 1.42 Ca	15.23 ± 1.40 Cb	12.51 ± 1.33 Cb	0.86 ± 0.70 Cc	0.78 ± 0.66 Bc	608.3	<0.01
	Maize	87.83 ± 1.01 Ba	31.21 ± 1.11 Ab	26.48 ± 1.42 Ab	19.68 ± 1.17 Ac	8.51 ± 1.08 Ad	694.8	<0.01
	Rice	103.93 ± 1.85 Aa	24.81 ± 1.78 Bb	19.91 ± 1.50 Bb	11.56 ± 1.15 Bc	0.80 ± 0.66 Bd	792.9	<0.01
	*F*	137	30.5	24.1	83.0	28.9		
	*p*	<0.01	<0.01	<0.01	<0.01	<0.01		
*T. castaneum*	Wheat	88.63 ± 0.89 Aa	28.03 ± 1.01 Bb	23.98 ± 1.43 Bb	16.61 ± 1.37 Bc	7.65 ± 0.96 Cd	763.1	<0.01
	Maize	64.06 ± 1.44 Ba	33.66 ± 0.84 Ab	29.13 ± 0.88 Abc	25.85 ± 1.31 Ac	18.16 ± 0.90 Ad	254.0	<0.01
	Rice	39.38 ± 1.41 Ca	20.05 ± 1.47 Cb	17.11 ± 1.46 Cbc	15.91 ± 1.20 Bbc	12.30 ± 1.36 Bc	58.9	<0.01
	*F*	371.1	35.6	21.8	18.2	23.1		
	*p*	<0.01	<0.01	<0.01	<0.01	<0.01		
*T. granarium*	Wheat	91.76 ± 0.54 Ba	34.43 ± 0.80 Cb	29.15 ± 0.88 Bc	21.10 ± 1.34 Bd	13.45 ± 1.31 Be	920.7	<0.01
	Maize	104.65 ± 1.83 Aa	57.15 ± 1.14 Ab	50.96 ± 1.30 Ac	41.56 ± 1.50 Ad	24.35 ± 1.32 Ae	433.4	<0.01
	Rice	73.61 ± 1.29 Ca	40.16 ± 1.76 Bb	31.20 ± 1.09 Bc	25.83 ± 1.31 Bc	18.06 ± 1.52 Bd	232.2	<0.01
	*F*	137.4	82.7	118.6	59.1	15.5		
	*p*	<0.01	<0.01	<0.01	<0.01	<0.01		
*R. dominica*	Wheat	113.98 ± 2.93 Aa	22.28 ± 1.36 Bb	16.51 ± 1.27 Bbc	9.86 ± 0.92 Bc	0.68 ± 0.52 Cd	806.2	<0.01
	Maize	44.66 ± 1.47 Ca	21.81 ± 1.03 Bb	19.18 ± 1.11 Bb	17.11 ± 0.92 Abc	12.65 ± 1.15 Ac	117.8	<0.01
	Rice	105.22 ± 2.32 Ba	33.65 ± 0.84 Ab	24.98 ± 1.54 Ac	15.18 ± 1.08 Ad	8.50 ± 1.08 Be	701.1	<0.01
	*F*	264.3	36.9	10.7	14.7	39.6		
	*p*	<0.01	<0.01	<0.01	<0.01	<0.01		

**Table 5:** ANOVA parameters of main effects and associated interaction for mortalities of *S. oryzae, T. castaneum, T. granarium*, and *R. dominica* adults after 7 days of exposure in laboratory persistence trials (total df = 143 for all species).

Species		*S. oryzae*		*T. castaneum*		*T. granarium*		*R. dominica*	
**Source**	**df**	***F***	***p***	***F***	***p***	***F***	***p***	***F***	***p***
Dose rate	3	234.52	<0.01	212.47	<0.01	155.41	<0.01	164.90	<0.01
Period of storage	3	1049.14	<0.01	913.53	<0.01	763.52	<0.01	698.05	<0.01
Dose rate × period of storage	9	10.20	<0.01	13.94	<0.01	15.81	<0.01	8.27	<0.01

**Table 6:** Mean mortality (% ± SE) of *S. oryzae, T. castaneum, T. granarium*, and *R. dominica* adults exposed on wheat treated with four dose rates of the combination of λ-cyhalothrin and chlorantraniliprole in four laboratory trials carried out from 0 to 90 days after treatment. For each species within each dose rate, means followed by the same lowercase letter are not significantly different; df = 3, 35, Tukey (HSD) test at *p* = 0.05. For each species within each period of storage, means followed by the same uppercase letter are not significantly different; df = 3, 35, Tukey (HSD) test at *p* = 0.05.

Species	Dose (ppm)	0 Days	30 Days	60 Days	90 Days	*F*	*p*
*S. oryzae*	0.01	52.74 ± 1.15 Da	34.37 ± 0.72 Cb	25.21 ± 1.81 Dc	7.93 ± 1.68 Cd	175.1	<0.01
	0.1	74.31 ± 2.35 Ca	51.54 ± 1.54 Bb	33.55 ± 1.17 Cc	12.47 ± 1.37 BCd	247.4	<0.01
	1	85.65 ± 2.14 Ba	57.00 ± 2.08 Bb	41.16 ± 1.79 Bc	18.05 ± 1.28 Bd	234.6	<0.01
	5	94.18 ± 0.98 Aa	68.71 ± 1.40 Ab	47.42 ± 1.19 Ac	24.63 ± 1.75 Ad	473.2	<0.01
	*F*	104.2	88.4	39.7	21.9		
	*p*	<0.01	<0.01	<0.01	<0.01		
*T. castaneum*	0.01	38.77 ± 1.17 Da	26.61 ± 1.46 Db	14.71 ± 0.95 Dc	0.00 ± 0.00 Cd	247.2	<0.01
	0.1	56.12 ± 1.55 Ca	38.20 ± 1.37 Cb	23.27 ± 1.68 Cc	5.80 ± 1.60 BCd	189.9	<0.01
	1	71.42 ± 1.74 Ba	45.38 ± 1.82 Bb	29.07 ± 1.83 Bc	8.54 ± 1.76 ABd	219.3	<0.01
	5	82.65 ± 1.80 Aa	53.59 ± 1.90 Ab	37.31 ± 1.01 Ac	12.65 ± 1.88 Ad	301.2	<0.01
	*F*	143.8	47.6	44.5	12.2		
	*p*	<0.01	<0.01	<0.01	<0.01		
*T. granarium*	0.01	31.97 ± 1.36 Da	18.77 ± 1.45 Cb	9.21 ± 1.36 Cc	0.00 ± 0.00 Bd	128.6	<0.01
	0.1	57.48 ± 1.22 Ca	24.21 ± 1.89 Cb	17.38 ± 1.93 Bc	0.00 ± 0.00 Bd	263.3	<0.01
	1	63.94 ± 1.63 Ba	35.48 ± 1.60 Bb	21.81 ± 2.04 Bc	3.41 ± 1.25 ABd	236.2	<0.01
	5	71.42 ± 1.97 Aa	46.75 ± 1.90 Ab	30.04 ± 2.24 Ac	7.52 ± 2.01 Ad	175.0	<0.01
	*F*	118.1	51.9	20.4	9.07		
	*p*	<0.01	<0.01	<0.01	<0.01		
*R. dominica*	0.01	45.75 ± 1.91 Da	32.51 ± 1.77 Db	21.28 ± 1.76 Cc	3.77 ± 1.45 Cd	105.8	<0.01
	0.1	62.13 ± 1.48 Ca	43.17 ± 1.62 Cb	29.17 ± 2.05 Bc	11.74 ± 1.51 Bd	161.3	<0.01
	1	76.78 ± 1.81 Ba	52.37 ± 2.03 Bb	35.04 ± 1.62 Bc	15.14 ± 1.50 ABd	221.0	<0.01
	5	87.35 ± 1.96 Aa	61.31 ± 2.18 Ab	42.94 ± 1.88 Ac	18.63 ± 1.71 Ad	222.6	<0.01
	*F*	100.0	41.3	24.8	16.8		
	*p*	<0.01	<0.01	<0.01	<0.01		

**Table 7:** ANOVA parameters of main effects and associated interactions for progeny production of *S. oryzae, T. castaneum, T. granarium*, and *R. dominica* individuals in laboratory persistence trials (total df = 179 for all species).

Species		*S. oryzae*		*T. castaneum*		*T. granarium*		*R. dominica*	
Source	df	*F*	*p*	*F*	*p*	*F*	*p*	*F*	*p*
Dose rate	4	2741.44	<0.01	2143.20	<0.01	2021.50	<0.01	2638.90	<0.01
Period of storage	3	150.40	<0.01	212.67	<0.01	209.29	<0.01	149.27	<0.01
Dose rate × period of storage	12	5.43	<0.01	4.57	<0.01	2.62	<0.01	4.75	<0.01

**Table 8:** Mean numbers (± SE) of *S. oryzae, T. castaneum, T. granarium*, and *R. dominica* individuals per vial following exposure of parents to wheat treated with four dose rates of the combination of λ-cyhalothrin and chlorantraniliprole and untreated control (0 ppm) in four laboratory trials carried out from 0 to 90 days after treatment. For each species within each treatment, means followed by the same lowercase letter are not significantly different; df = 3, 35, Tukey (HSD) test at *p* = 0.05. For each species within each period of storage, means followed by the same uppercase letter are not significantly different; df = 4, 44, Tukey (HSD) test at *p* = 0.05.

Species	Dose (ppm)	0 Days	30 Days	60 Days	90 Days	*F*	*p*
*S. oryzae*	0	81.28 ± 1.91 Ab	86.51 ± 2.20 Ab	93.23 ± 1.32 Aa	99.06 ± 0.71 Aa	22.4	<0.01
	0.01	8.45 ± 1.88 Bc	21.88 ± 1.27 Bb	27.01 ± 1.65 Bb	35.16 ± 1.57 Ba	48.3	<0.01
	0.1	0.45 ± 0.14 Cd	7.06 ± 1.52 Cc	15.33 ± 1.36 Cb	21.50 ± 1.72 Ca	47.4	<0.01
	1	0.53 ± 0.18 Cc	0.33 ± 0.16 Dc	8.26 ± 1.51 Db	13.46 ± 1.81 Da	28.7	<0.01
	5	0.48 ± 0.20 Cb	0.38 ± 0.16 Db	4.05 ± 1.35 Db	9.25 ± 1.51 Da	16.5	<0.01
	*F*	856.7	751.3	639.9	583.4		
	*p*	<0.01	<0.01	<0.01	<0.01		
*T. castaneum*	0	94.58 ± 2.41 Ac	101.07 ± 1.93 Abc	106.52 ± 1.85 Aab	113.98 ± 1.67 Aa	17.1	<0.01
	0.01	13.65 ± 1.45 Bd	32.21 ± 1.49 Bc	38.73 ± 2.04 Bb	45.83 ± 1.35 Ba	73.4	<0.01
	0.1	5.11 ± 2.16 Cd	16.41 ± 1.55 Cc	27.90 ± 1.33 Cb	36.16 ± 2.40 Ca	50.1	<0.01
	1	0.25 ± 0.06 Cd	9.68 ± 1.50 CDc	21.76 ± 1.51 Cb	28.41 ± 1.85 Da	78.6	<0.01
	5	0.40 ± 0.10 Cc	3.13 ± 1.87 Dc	8.85 ± 1.44 Db	17.73 ± 1.56 Ea	29.1	<0.01
	*F*	649.3	558.7	532.9	444.1		
	*p*	<0.01	<0.01	<0.01	<0.01		
*T. granarium*	0	99.35 ± 2.96 Ac	107.95 ± 2.34 Ab	114.87 ± 1.27 Aab	117.43 ± 1.05 Aa	15.4	<0.01
	0.01	21.41 ± 1.69 Bc	38.16 ± 1.64 Bb	46.81 ± 1.66 Ba	53.15 ± 1.50 Ba	71.5	<0.01
	0.1	12.93 ± 1.55 Cc	23.88 ± 1.49 Cb	35.50 ± 2.54 Ca	41.11 ± 1.65 Ca	45.3	<0.01
	1	6.45 ± 1.53 CDd	14.38 ± 1.30 Dc	24.21 ± 1.32 Db	35.61 ± 2.51 Ca	52.3	<0.01
	5	0.66 ± 0.52 Dd	9.70 ± 1.49 Dc	16.15 ± 1.52 Eb	23.86 ± 1.49 Da	54.5	<0.01
	*F*	492.2	561.7	518.9	462.5		
	*p*	<0.01	<0.01	<0.01	<0.01		
*R. dominica*	0	97.80 ± 1.95 Ab	102.57 ± 2.15 Ab	104.87 ± 1.75 Ab	115.18 ± 1.75 Aa	14.7	<0.01
	0.01	11.93 ± 1.79 Bc	29.51 ± 1.31 Bb	33.20 ± 2.15 Bb	41.61 ± 1.77 Ba	48.9	<0.01
	0.1	3.13 ± 1.32 Cd	12.95 ± 1.56 Cc	20.95 ± 1.67 Cb	28.48 ± 1.85 Ca	45.1	<0.01
	1	0.85 ± 0.65 Cc	5.18 ± 1.08 Dc	12.98 ± 1.57 Db	19.81 ± 1.91 Da	36.5	<0.01
	5	0.88 ± 0.70 Cc	0.98 ± 0.64 Dc	7.11 ± 1.51 Db	13.11 ± 1.61 Da	23.5	<0.01
	*F*	909.7	840.2	518.4	537.9		
	*p*	<0.01	<0.01	<0.01	<0.01		

## Data Availability

The original contributions presented in this study are included in the article. Further inquiries can be directed to the corresponding authors.
